# Initial Symptoms of Langerhans Cell Histiocytosis: A Case Series

**DOI:** 10.1177/2333794X19857377

**Published:** 2019-06-19

**Authors:** Yukari Atsumi, Yuya Saito, Hiroshi Hataya, Yuki Yuza

**Affiliations:** 1 Department of General Pediatrics, Tokyo Metropolitan Children’s Medical Center, Tokyo, Japan; 2 Department of Hematology and Oncology, Tokyo Metropolitan Children’s Medical Center, Tokyo, Japan

**Keywords:** Langerhans cell histiocytosis, initial symptom, bone lesion

## Abstract

Langerhans cell histiocytosis (LCH) is a rare childhood hematopoietic disease, and hence, there are few reports summarizing the course leading to the diagnosis. We described the initial symptoms and the clinical course of LCH. We carried out a retrospective review of charts from a single medical center, and 21 patients with the diagnosis of LCH were enrolled. The initial symptoms of 16 cases were caused by bone lesions; of these cases, there were 8 instances of soft tissue swelling as the initial symptom (38%) and 8 instances of bone pain without swelling (38%). Among the cases of bone lesion, 4 of 6 cases of skull lesion were painless while all vertebral body lesions and long bone lesions were accompanied by pain. LCH bone lesions caused various symptoms depending on the site of the lesion and this makes the diagnosis difficult. A detailed physical examination and imaging studies are recommended for early diagnosis.

## Introduction

Langerhans cell histiocytosis (LCH) is a rare hematopoietic disease with 60 to 70 pediatric cases reported per year in Japan, recently linked to BRAF gene mutation.^1^ It has been suggested that LCH is a neoplastic disease. However, unlike in typical neoplastic diseases, the lesions in LCH do not metastasize but continue to remain at the initial lesion site. Although the prognosis may differ between the lesion sites, it is generally good.^2,3^ Craniofacial bone lesions, such as those of the temporal, orbital, mastoid antrum, cheekbones, upper jaw, and paranasal sinuses, are considered to be risk factors for central nervous involvement, and patients with such lesions also frequently have central diabetes insipidus (DI). There are as yet no reports on the efficacy of early diagnosis for LCH patients except in cases of central DI, which is thought to be preventable by early treatment.^4,5^ However, the timing of diagnosis remains a serious issue for patients, and early treatment may influence their functional outcomes.

LCH lesions occur most commonly in the bone and can develop in any bone in the body. A previous study showed that 57% of patients presented with localized bone lesions.^6^ There are numerous reports of bone lesions in LCH,^6-9^ and various symptoms of bone lesions are known. However, few have detailed the initial symptoms or the physical examinations of bone lesions performed. The initial symptoms are those that are noticed first by the patients, caregivers, or medical staff and lead to the patients’ admittance to a hospital. LCH involves various sites in the body, thus, LCH patients present with various types of symptoms and may, therefore, be examined first in any of a number of medical departments including pediatrics, orthopedics, and otolaryngology. This fact is important to remember for detecting the initial symptom of this disease in its early stages.

We describe here the initial symptoms of LCH patients. We collected data pertaining to initially symptoms, physical examination findings, and symptoms that led patients to seek medical attention with the aim of clarifying the course of the disease for early diagnosis.

## Methods

### Study Design

The present study was designed as a retrospective case series study using the database at Tokyo Metropolitan Children’s Medical Center and Tokyo Metropolitan Kiyose Children’s Hospital between April 1995 and August 2015. The Tokyo Metropolitan Kiyose Children’s Hospital was closed in 2010. Four hospitals including Tokyo Metropolitan Kiyose Children’s Hospital were integrated, and Tokyo Metropolitan Children’s Medical Center was opened in March 2010. We enrolled LCH patients who received a biopsy and were definitively diagnosed at either of the 2 hospitals.

We collected data on age, sex, initial symptoms, time to diagnosis from initial symptoms, lesion site (skin, bone, lymph nodes), treatment history, blood test date, diagnostic imaging findings, survival, and the presence of complications if any. The patients were divided into single site (SS) group and multiple sites (MS) group. The SS group was then divided into an SS-s group, in which only one lesion occurred in a single organ, and the SS-m group, in which multiple lesions occurred in a single organ.

The time of diagnosis was defined as the day of the biopsy. Bone lesions with the adjacent soft tissue were defined as a single organ lesion. The initial symptoms or lesion sites were defined as the symptoms or lesion sites first noticed by patients, their family members, or the medical personnel. A complication was defined as an irreversible disease that required the patients to continue receiving medical attention at the time to be included in the study.

We classified the symptoms into 10 groups as follows: (1) Soft tissue swelling (bone lesions); (2) bone pain without swelling (bone lesions); (3) polydipsia or polyuria (DI); (4) rash (skin); (5) abdominal distension (splenohepatomegaly); (6) malaise (due to hematopoietic abnormalities); (7) cough or respiratory discomfort (lung); (8) lymph node swelling; (9) central nervous system manifestations; and (10) other.

### Data Analysis

We performed a descriptive analysis of the collected data. In addition, we compared the days from the appearance of the initial symptoms to diagnosis between groups according to the initial symptoms or the types of LCH. The Mann-Whitney *U* test was used for continuous variables, and *P* < .05 was considered statistically significant. All statistical analyses were carried out using the IBM SPSS statistics software for Windows 22.0.0.

### Ethical Approval and Informed Consent

The study was planned in accordance with the Declaration of Helsinki and was approved by the institutional review board. Written or verbal informed consent from this study population is not required because this is a retrospective study and there is no intervention to the patient. Information was released by posting documents approved by the review board in Tokyo Metropolitan Children’s Medical Center.

## Results

During the study period, 23 patients were registered as new LCH patients, and 2 patients were excluded because they were diagnosed or treated at another hospital. Finally, 21 patients were enrolled. Among these, 6 patients (29%) were allocated to the SS-s group, 3 patients (14%) were allocated to the SS-m group, and 12 patients (57%) were allocated to the MS group. The median age of the patients at diagnosis was 2.8 years (range = 0.1-6.7 years), and 14 patients (67%) were male. The initial blood test date showed an increased level of lactate dehydrogenase, ferritin, and soluble IL-2 receptor (sIL-2r) as in previous reports. The median time to diagnosis from the initial symptoms was 44 days (range = 11-1566 days). Nineteen patients (90%) were treated with chemotherapy, and there were no deaths. Nine patients experienced complications, 4 patients had a compression fracture, 3 patients had DI, and 2 patients showed lung involvement (Table 1).

**Table 1.** Patient Characteristics.

Characteristic	
Age (years), median (range)	2.8 (0.1-6.7)
Sex, male (%)	14/21 (67)
Initial blood data, median (range)	
WBC (/µL)	9570 (4460-24 020)
Hb (mg/dL)	11.8 (4.0-13.8)
Plt (10^4^/µL)	43.5 (1.1-74.5)
AST (IU/L)	26 (19-162)
ALT (IU/L)	11 (8-57)
LDH (IU/L)	223 (163-685)
CRP (mg/dL)	0.89 (0.05-5.12)
Ferritin (ng/mL)	23.5 (0.16-1176.5)
IL-2r (U/mL)	778 (294-9500)
Type of LCH	
SS-s	6/21 (29)
SS-m	3/21 (14)
MS	12/21 (57)
Time to diagnosis from an initial symptom (days), median (range)	42 (11-156)
Treatment	
Chemotherapy (%)	19/21 (90)
Only curettage (%)	2/21 (10)
Outcome	
Death (%)	0/21 (0)
Complications (%)	9/21 (43)

Abbreviations: WBC, white blood cell; Hb, hemoglobin; Plt, platelet; AST, aspartate aminotransferase; ALT, alanine aminotransferase; LDH, lactate dehydrogenase; CRP, C-reactive protein; IL-2, interleukin-2; SS-s, 1 lesion in a single organ; SS-m, multiple lesions in a single organ; MS, multiple lesions in multiple organs.

**Figure 1.**
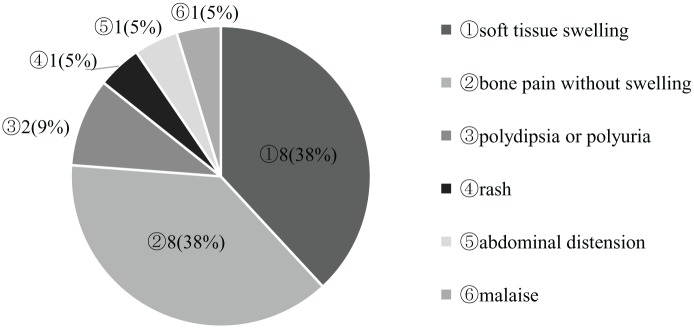
Initial symptoms (n = 21). Most initial symptoms were bone lesions. There were 8 cases of soft tissue swelling (38%) and 8 cases of bone pain (38%). Malaise was due to hematopoietic abnormalities stemming from anemia.

Most initial symptoms originated from bone lesions, soft swelling associated with lesions, and bone pain (Figure 1). Two of 8 patients with soft tissue swelling had associated pain, origination from skull lesions. Among the patients with bone lesions as the initial symptoms, 4 out of 6 patients with a skull lesion reported no pain, whereas all patients with vertebral body or long bone lesions reported pain (Table 2). The time to diagnosis from the appearance of the initial symptoms was shorter in patients reporting pain than in those who did not, but the difference was not significant. Longest duration between appearances of initial symptom to medical consultation was noted in a patient who presented initially with polydipsia due to central DI secondary to a painless skull lesion. At the time of diagnosis, only one patient did not have a bone lesion.

**Table 2.** Cases With Bone Lesion as Initial Symptoms^a^.

	Painful		Painless	
Swelling	Skull	2	Skull	4
			Scapula	1
			Sternum	1
Not swelling	Vertebral body	3		
	Femur	2		
	Humerus	1		
	Ischium	1		
	Ilium	1		
Time to diagnosis from an initial symptom (days), median (range)*	28 (11-172)		45 (20-1566)	

* *P* = .057, Mann-Whitney *U* test.

a Sixteen cases presented to the hospital with bone lesions as initial symptoms. Two of 8 patients (25%) with soft tissue swelling had pain. The time to diagnosis from an initial symptom in painful is shorter than in painless, but there is no significant difference. Four out of 6 (66%) patients with skull lesion was painless, and all patients with long bones and vertebral body lesions were painful. Eight of 10 cases (80%) with painful lesions did not have the physical findings including swelling.

All of the patients in the SS group had bone lesions. Five of 12 patients in the MS group (42%) presented with initial symptoms unrelated to bone. Among MS group patients, 2 presented with DI while other had anemia, exanthema, or hepatosplenomegaly. In the MS group presenting with initial symptoms unrelated to bone, the median period from the appearance of the initial symptom to diagnosis was 87 days (range = 12-137 days). There is no significant difference between time to diagnosis of MS type and SS type. The shortest duration between appearance of initial symptom and diagnosis was observed in a patient who had hepatosplenomegaly which suddenly deteriotrated.

## Discussion

Our investigation of the initial symptoms of LCH showed that bone lesions were common especially among children with LCH, and as mentioned above, the present study showed that more than 70% of patients presented with initial symptoms related to bony lesions. Although MS-type cases are known to have variable symptoms,^10,11^ over half of such cases had bone lesions as the initial symptoms in the present study, suggesting that bone lesions develop in LCH patients from an early stage.

In this study, patients diagnosed with LCH were divided into 2 groups—SS and MS groups, and those in the latter group presenting with nonspecific symptoms made it challenging to diagnose.^10,11^ In the present study, longest duration from presentation to diagnosis was noted in patients with symptoms related to bony lesions. On the other hand, patients in the MS group deteriorated rapidly that led to earlier evaluation and diagnosis.

Bone lesions in LCH led to osteolytic changes and pain, but some cases with painless soft tissue swelling have also been reported. In the present study, in patients with bone lesions as the initial symptoms, most of the painful lesions were undetectable by physical examination, and the swollen lesions tended to be painless. Bone lesions were not identified by physical examination in patients presenting with bone pain but were detected by imaging studies. Although there is no statistically significant difference, the present study reveals that painless lesions are associated with longer duration to diagnosis than painful lesions. LCH patients presenting with central DI have been reported.^11^ In our study, there was 1 patient who presented with polydipsia due to central DI secondary to painless skull lesions.

There are only few reports discussing the lesion sites and pain in LCH.^9,12^ It is unclear as to the reason behind painful versus painless lesions in LCH. In children with leukemia, bone pain is a common symptom, and the long bones and vertebral bodies are common loci of pain, which is thought to be caused by the massive proliferation of hematopoietic tissue within the medullary cavities,^13,14^ although the reason is still unclear. Painful bone lesions are often reported in the ribs, femur, and spine.^7^ The present study showed that more than half of the patients with bone lesions reported pain, and that all vertebral body and long bone lesions were painful. These results suggest that loaded bones and highly mobile bones are most likely to be loci of pain. The previous studies showed that most of the patients with skull lesions did not complain of pain.^15,16^ However, one study reported that some patients at presentation had a painful bone lesion including a skull lesion^12^ but omitted any further details about the skull lesion. The present study showed that there were only 2 painful skull lesions, one in the butterfly bone and the other in the mandible. These results suggest that among skull lesions, infectious lesions and lesions in highly mobile bones may become painful.

This study has 2 strengths. First, the study was done in a single center, thus, it was possible to search for detailed information on the locus of pain and the sites of the lesions at the time of examination and diagnosis. Our search failed to uncover any reports focusing on the difference in lesion sites or physical findings between painful and painless lesions. Second, the study facility is the chief children’s hospital in the region. It was, therefore, possible to study the long-term prognosis without patients dropping out. Both medical and surgical complications were followed-up at our hospital, enabling us to clarify the course of the disease.

This study has 3 limitations. First, this was a retrospective study, thus, we were limited to the symptoms and physical examinations described in the medical records. It is sometimes difficult for physicians to evaluate pain experienced by young children, and the assessment might also differ depending on the evaluators. Second, the follow-up periods differed among the patients, thus, complications and progression over the long term could not be assessed. Third, the research was done at a single facility, and therefore, the generalizability of the results is uncertain. The follow-up and complications also may not be generalized.

## Conclusion

Most LCH patients will have bone lesions during their initial presentation, which may vary depending on the site of lesion. This makes diagnosis difficult, and hence, detailed physical examination and appropriate imaging studies are recommended for early diagnosis.
